# Rice bran extract (RBE) as supplement for cell culture

**DOI:** 10.1186/1753-6561-7-S6-P106

**Published:** 2013-12-04

**Authors:** Satoko Moriyama, Ken Fukumoto, Masayuki Taniguchi, Shigeru Moriyama, Takuo Tsuno, Satoshi Terada

**Affiliations:** 1 University of Fukui, Fukui, 910-8507, Japan; 2 Niigata University, Niigata, 950-2102, Japan; 3 Tsuno Food Industrial Co., Ltd, Katsuragi-cho, Wakayama, 649-7122, Japan

## Introduction

In mammalian cell culture, fetal bovine serum (FBS) or proteins obtained from mammals are usually supplemented to culture media. Since the use of animal-derived components may cause an infection of virus and other pathogens, alternative supplement derived from non-mammals is eagerly required in cell culture for producing biotherapeutics and for cell therapy [1]. As an alternative supplement, we focused on rice bran extract (RBE), because several studies have been done and reported that RBE has some biological effects such as enhancement of NK cell activity and anti-inflammatory effect on mice [2] and antioxidant effect [3].

Rice bran, by-product of milling in the production of refined white rice, contains abundant nutrients and proteins. In this study, the effect of RBE was examined in the serum-free culture.

## Materials and methods

### Effect of RBE on several cell lines

RBE was extracted from rice bran in an alkaline solution, precipitated with acid, and subsequently freeze-dried. The proceeding was performed by Tsuno Food Infdustrial Co., Ltd. To test the effect, RBE was supplemented to the culture of hybridoma cells, Chinese hamster ovary cells (CHO-DP12), hepatoma HepG2 and HeLa. The cells were cultured in 24 well plate (Sumitomo Bakelite, Japan) with 1 ml ASF104 medium (Ajinomoto, Japan) containing RBE or BSA (Wako, Japan) as positive control. The cell density was estimated using a hemacytometer. Viable cells were distinguished from dead cells by trypan blue dye exclusion method. The production of antibodies from hybridoma and CHO-DP12 cell was measured by ELISA method.

### Fractionation of RBE with UF membrane

In order to identify the growth factor(s) in RBE, fractionations were performed using UF membranes. RBE was fractionated into the permeable and residual fraction with ultrafiltration membrane Amicon Ultra-15 (Merck Millipore, Germany) at 4,000 rpm, 40 min and 4°C. The fractions and whole RBE were added to the culture of hybridoma and CHO-DP12 cells.

## Results and discussion

### Enhanced cell growth and productivity using RBE

**Figure 1:**
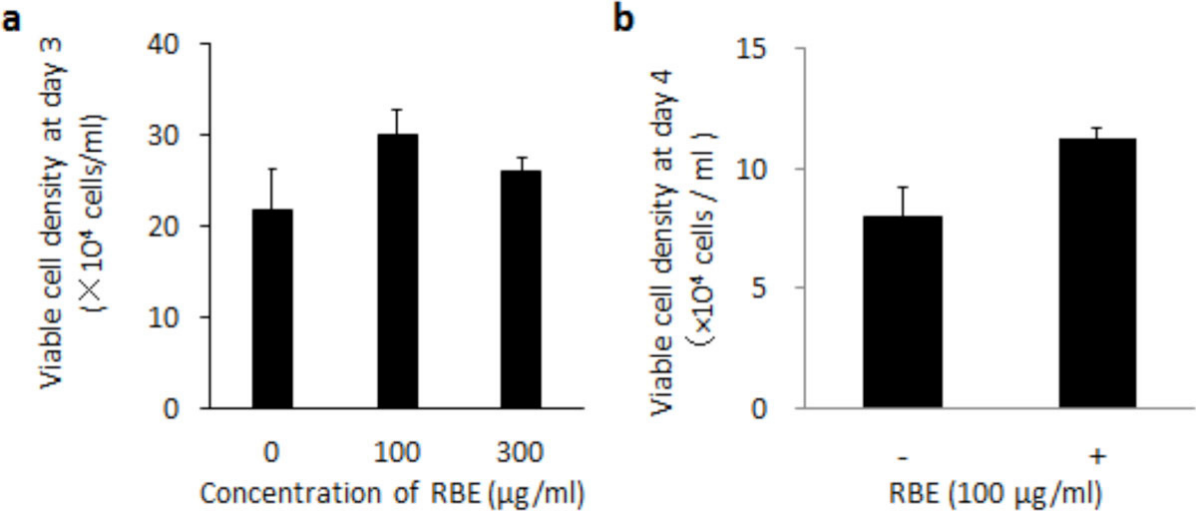
**Cell growth in serum-free medium containing RBE**. **a **mouse hybridoma cell, **b **HeLa.

Figure 1 shows an enhanced proliferation by RBE. On growth and monoclonal antibody production of hybridoma cells, RBE had desired effect and the effect of RBE was superior to that of BSA. Similarly, to CHO-DP12 cells, addition of RBE exhibited increased cell growth and improved the productivity of humanized antibody. Growth of HepG2 and HeLa cells were also enhanced in the presence of RBE.

### Improvement of fractionated RBE by UF membrane

Fractionated RBEs by UF membranes were also tested. The fraction of RBE more than 60 kDa improved the proliferation of hybridoma cells and the level was superior to that of whole RBE, while the fraction less than 60 kDa inhibited the proliferation. This results suggest that in RBE, some lower molecular inhibitor(s) and higher molecular growth factor(s) would be contained.

## Conclusion

We provide the first evidence that RBE is an attractive culture supplement to improve the proliferation and the production of mammalian cells.
